# *In situ* theranostic platform combining highly localized magnetic fluid hyperthermia, magnetic particle imaging, and thermometry in 3D

**DOI:** 10.7150/thno.86759

**Published:** 2024-01-01

**Authors:** Oliver Buchholz, Kulthisa Sajjamark, Jochen Franke, Huimin Wei, André Behrends, Christian Münkel, Cordula Grüttner, Pierre Levan, Dominik von Elverfeldt, Matthias Graeser, Thorsten Buzug, Sébastien Bär, Ulrich G. Hofmann

**Affiliations:** 1 Section for Neuroelectronic Systems, Department of Neurosurgery, University Medical Center Freiburg, Faculty of Medicine, University of Freiburg, Freiburg, Germany.; 2 Bruker BioSpin MRI GmbH, Preclinical Imaging Division, Ettlingen, Germany.; 3 Fraunhofer Research Institution for Individualized and Cell-Based Medical Engineering IMTE, Lübeck, Germany.; 4 Micromod Partikeltechnologie GmbH, Rostock, Germany.; 5 Department of Radiology and Hotchkiss Brain Institute, Cumming School of Medicine, University of Calgary, Calgary, AB, Canada.; 6 Division of Medical Physics, Department of Diagnostic and Interventional Radiology, University Medical Center Freiburg, Faculty of Medicine, University of Freiburg, Freiburg, Germany.; 7 Institute of Medical Engineering, University of Lübeck, Germany.

**Keywords:** Magnetic Particle Imaging, Magnetic Fluid Hyperthermia, Theranostic, Thermometry, Imaging

## Abstract

Theranostic platforms, combining diagnostic and therapeutic approaches within one system, have garnered interest in augmenting invasive surgical, chemical, and ionizing interventions. Magnetic particle imaging (MPI) offers a quite recent alternative to established radiation-based diagnostic modalities with its versatile tracer material (superparamagnetic iron oxide nanoparticles, SPION). It also offers a bimodal theranostic framework that can combine tomographic imaging with therapeutic techniques using the very same SPION.

**Methods**: We show the interleaved combination of MPI-based imaging, therapy (highly localized magnetic fluid hyperthermia (MFH)) and therapy safety control (MPI-based thermometry) within one theranostic platform in all three spatial dimensions using a commercial MPI system and a custom-made heating insert. The heating characteristics as well as theranostic applications of the platform were demonstrated by various phantom experiments using commercial SPION.

**Results**: We have shown the feasibility of an MPI-MFH-based theranostic platform by demonstrating high spatial control of the therapeutic target, adequate MPI-based thermometry, and successful *in situ* interleaved MPI-MFH application.

**Conclusions**: MPI-MFH-based theranostic platforms serve as valuable tools that enable the synergistic integration of diagnostic and therapeutic approaches. The transition into *in vivo* studies will be essential to further validate their potential, and it holds promising prospects for future advancements.

## Introduction

Diagnosis and therapy are two sides of the same coin applied by medical professionals. Typically, they are executed asynchronously, but advancements in technology have made it feasible to bring the coin back into one palm, leading to the creation of the neologism 'theranostics' 1,2. Although it originally aimed to explain the dual use of substances e.g. in nuclear medicine, point of care diagnostic and drug delivery 3, theranostics does propose instrumentation capable of both imaging and treatment in one device 4.

The foundational concept of MPI was introduced in 2005 entailing an alternating magnetic field (drive field), which leads to a change of the magnetization of superparamagnetic iron oxide nanoparticles (SPION) 5. MPI is a tomographic imaging technique which enables non-invasive quantification of versatile SPION 6,7. The intrinsic superparamagnetic properties of these SPION are exploited, as their non-linear magnetization response results in a distorted signal which can be detected by a receive system. The induced SPION signal, analyzed in the frequency domain, contains a plurality of harmonics of the drive frequency 8. This induced signal scales linearly with the SPION concentration 9 and thus makes MPI a quantitative method. By superimposing a static magnetic gradient field (selection field) vanishing at the center (field free region, FFR), the SPION signal can be encoded in space to permit for tomographic imaging 5. This is a consequence of the SPIONs' magnetization curve as they magnetically saturate outside the FFR and therefore do not contribute to the received signal 5. In contrast, SPION inside the FFR contribute distinctively to the received signal and thus permit tomographic image formation 5. During image acquisition, the FFR is moved in space by three superimposed magnetic fields (drive fields) in a predefined trajectory spanning an accessible field of view (FOV) 8,10. Extending or shifting the FOV can be realized by additional quasi-static magnetic fields (focus fields).

Since only the SPIONs' specific magnetization properties contribute to the image signals, MPI displays high sensitivity and a background free contrast 11. *In vivo* SPION remain superparamagnetic until hydrolyzed, enzymatically degraded 12 or excreted along the mononuclear phagocytic system 13,14 offering the possibility for long term monitoring depending on their metabolic properties 15. Also, changes in the SPION's environment such as its temperature, viscosity, and pH 16-18 influence their magnetization behavior. This opens the door for a wide range of applications 19 including MPI-based thermometry 20.

While contributing to image generation in the FFR, SPION may become subject to further physical manipulations. For example, high frequency alternating magnetic fields (AMF) may lead to energy dissipation (heating) caused by magnetization losses governed by internal Néel fluctuations of (single domain) SPION magnetic moments and external Brownian fluctuations 21. This AMF induced SPION heating is called magnetic fluid hyperthermia (MFH) 22. By exploiting the same spatial encoding mechanism as in MPI, the volume in which SPION contribute to MFH can be confined in 3D by the same magnetic gradient field to the vicinity of the FFR 23. Heating efficiency is determined by SPION's specific-absorption rate (SAR) and is dependent on shape anisotropy 24, coating material 25 and thickness 26, particle size 27, and thermal conductivity of the surface coating and solvent viscosity 28. From an instrumentation point of view, the SAR is governed by the amplitude and frequency of the AMF excitation while the spatial specificity is dominated by the magnetic field gradient slope. Additionally, the specificity is a function of the magentization response i.e., the point spread function of the SPIONs.

Combining magnetic particle imaging and MFH offers unprecedented benefits for the field of theranostics and was indeed already attempted in back-to-back instruments to selectively target liver tumors *in vivo* without substantially heating surrounding tissue 29.

In the study at hand, we go beyond this success and demonstrate tomographic MPI interleaved with locally restricted MFH (MPI-MFH) while controlling for achieved temperatures with MPI-based thermometry within the same platform and session. In several phantom experiments, we demonstrate the spatial control of the therapeutic target which enables precise MFH application at any position within the FOV of the introduced platform. Further, the MFH performance of several commercial SPION types is investigated and the feasibility of interleaved MPI-MFH experiments with combined MPI-based thermometry is shown. The MPI-MFH platform features a FFR that enables versatile MPI and MFH targeting in every direction.

The aim of the study was to introduce the first integrated MPI-MFH platform capable of application in 3D.

## Results

### MPI system with integrated AMF insert enables SPION based MFH

In this study, an *in situ* theranostic platform was established allowing for interleaving diagnostics with magnetically focused MFH-therapy and MPI-based temperature monitoring without the need for target relocation. The platform consists of a commercial preclinical MPI system (MPI 25/20FF, Bruker BioSpin MRI GmbH, Ettlingen) as a tomographic imaging device equipped with a custom-made hyperthermia insert 30 serving as a therapeutic tool.

**Figure 1:**
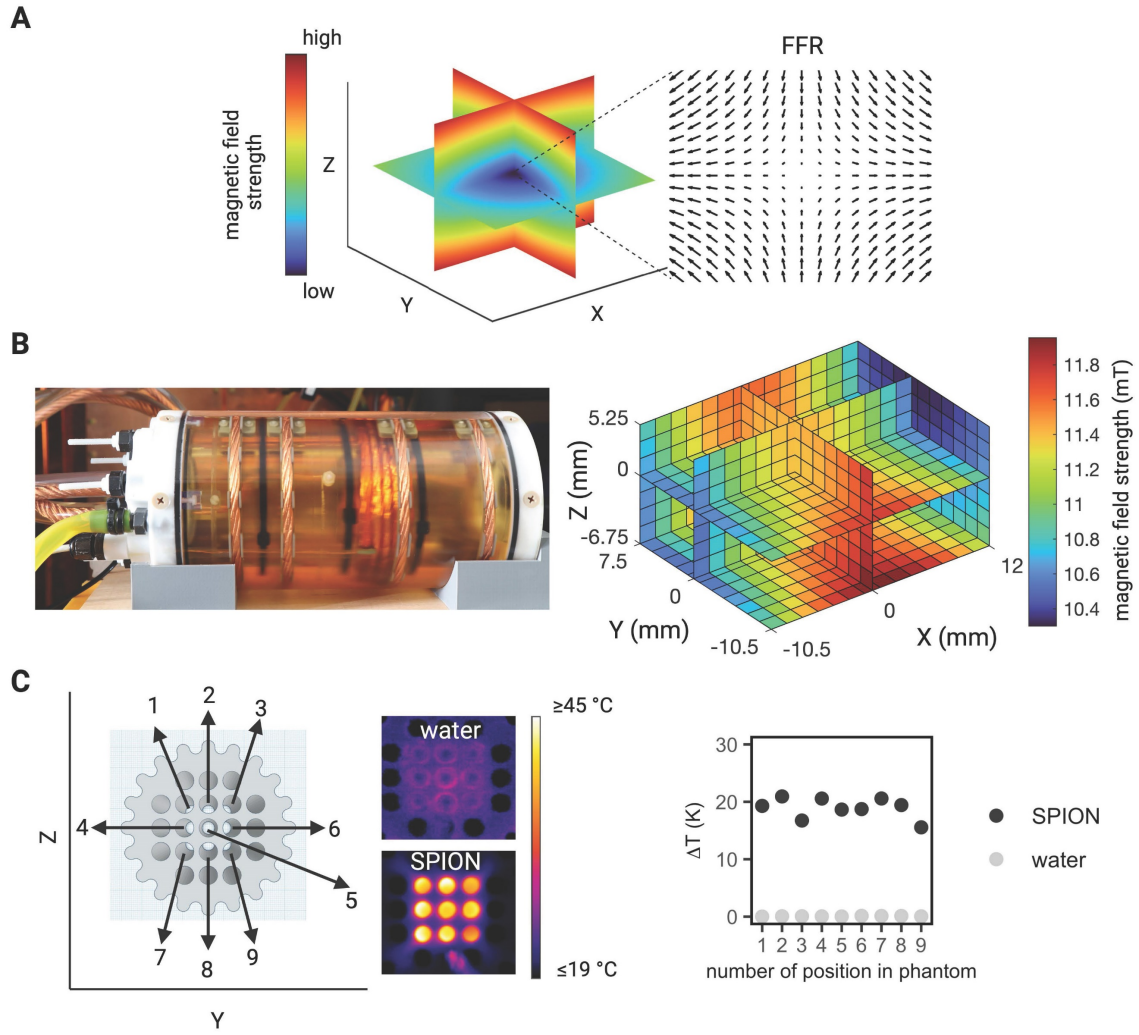
**MPI-guided MFH. (A)** Visualization of the gradient field distribution in space resulting from spatially varying magnetic fields in an MPI system. Only SPION located in the region with a vanishing magnetic strength (field free region (FFR) at the center) can interact with additional magnetic influences (e.g., imaging and heating). **(B)** Photograph of the heating insert (left) and the calculated magnetic field strength at the center of the hyperthermia insert over a volume of 22.5 mm x 18 mm x 12 mm (X, Y, Z) (right). **(C)** Proof of concept characterization of global MFH application (selection field OFF) in a 3D phantom of SPION samples within the field of therapy (FOT) distributed over a grid of 16 mm x 16 mm (in Y- and Z- direction) surrounding the center of the MPI-MFH platform (X = 0) using a thermal camera.

The MPI system generates a magnetic field gradient of up to 2.5 T/m in the Z- direction and 1.25 T/m in the X- and Y- directions (selection field) used for both spatial encoding during imaging and definition of the MFH target. By means of 3 orthogonal homogeneous magnetic offset fields (focus fields) with amplitudes of up to 17 mT, 17 mT and 42 mT in the X-, Y-, and Z- directions, respectively (see Figure 1A for a representative overview), both the imaging FOV and the field of therapy (FOT) can be shifted in space. During MPI image generation, the SPION are excited using 3 time-variant orthogonal homogeneous magnetic fields (drive fields) with amplitudes of up to 14 mT in each direction and resonance frequencies near 25 kHz. For heat generation during MFH application, the SPION are excited using a custom-made single-axis time-variant homogeneous magnetic field (hyperthermia field) applied by a hyperthermia insert 30 which was designed as a gradiometric coil to decouple it from the transmit-receive drive field coil set (for imaging). The hyperthermia insert is placed concentrically inside the MPI's bore. A radio frequency power amplifier (T & C Power Conversion, Inc. Model AG 1012 Amplifier Generator) was used to generate and amplify the signal of the system. When applying the maximum working power of 600 W, the hyperthermia insert is capable of generating an average field strength of 11.2 mT, with a standard deviation of 4 x 10^-4^ mT and a percentage homogeneity of the AMF field of -8.5%, 3.25%, 1.5% (for X, Y, Z) over a volume of 22.5 mm x 18 mm x 12 mm (for X, Y, Z) (see Figure 1B for a representative overview). The MFH field strength for all measurements presented in this work was set to 10 mT along the X-axis. In order to limit the power transferred to the MPI scanner, the hyperthermia insert has cancellation winding which decouples the hyperthermia coil from the drive field and receive coils 30. The hyperthermia insert itself is cooled with oil to avoid hardware overheating and additionally an airflow system was applied at the MPI bore entry. This was of systemic importance for achieving maximal heating durations and steady temperatures of the targeted SPION, while minimizing the risk of overheating. As the MFH-excitation frequency of 715 kHz lays within the imaging detection bandwidth of the MPI system, its low noise receive amplifiers were safeguarded with an additional low-pass filter and amplifier-blanking device. While the low-pass filter suppresses not only the MFH feed-through-signal but also the high frequency components of the imaging signal, the usage of an amplifier blanking has no negative impact to the imaging bandwidth but needs active components which interact with the theranostic scan program. With the hyperthermia insert installed, the free accessible bore diameter of the integrated theranostic platform measures 65 mm, suitable for preclinical *in vivo* studies of small rodents up to rat-sized animals.

A powerful user-interface accesses the hardware communication and signal generation for the entire theranostic platform. The user-interface allows the user to define all imaging and heating parameters, enabling selection of a therapy volume based on existing images.

Before assessing the whole integrated MPI-MFH platform, the general capacity of MFH within the FOV was tested. SPION suspensions and water samples of the same volume were subjected to MFH and the temperature profile was monitored with a thermal camera (FLIR Systems) (see Figure 1C). Depending on the position within the sample holder, and therefore the relative position within the MPI-MFH platform, temperature increases of 15.6 K and up to 20.9 K were observed for aqueous SPION samples. In the control samples without any SPION, only negligible heating (up to 0.1 K) was observed. We therefore conclude that the source of the detected SPION suspension heating was caused by the AMF induced MFH and did not result from system or phantom related heating effects.

**Figure 2:**
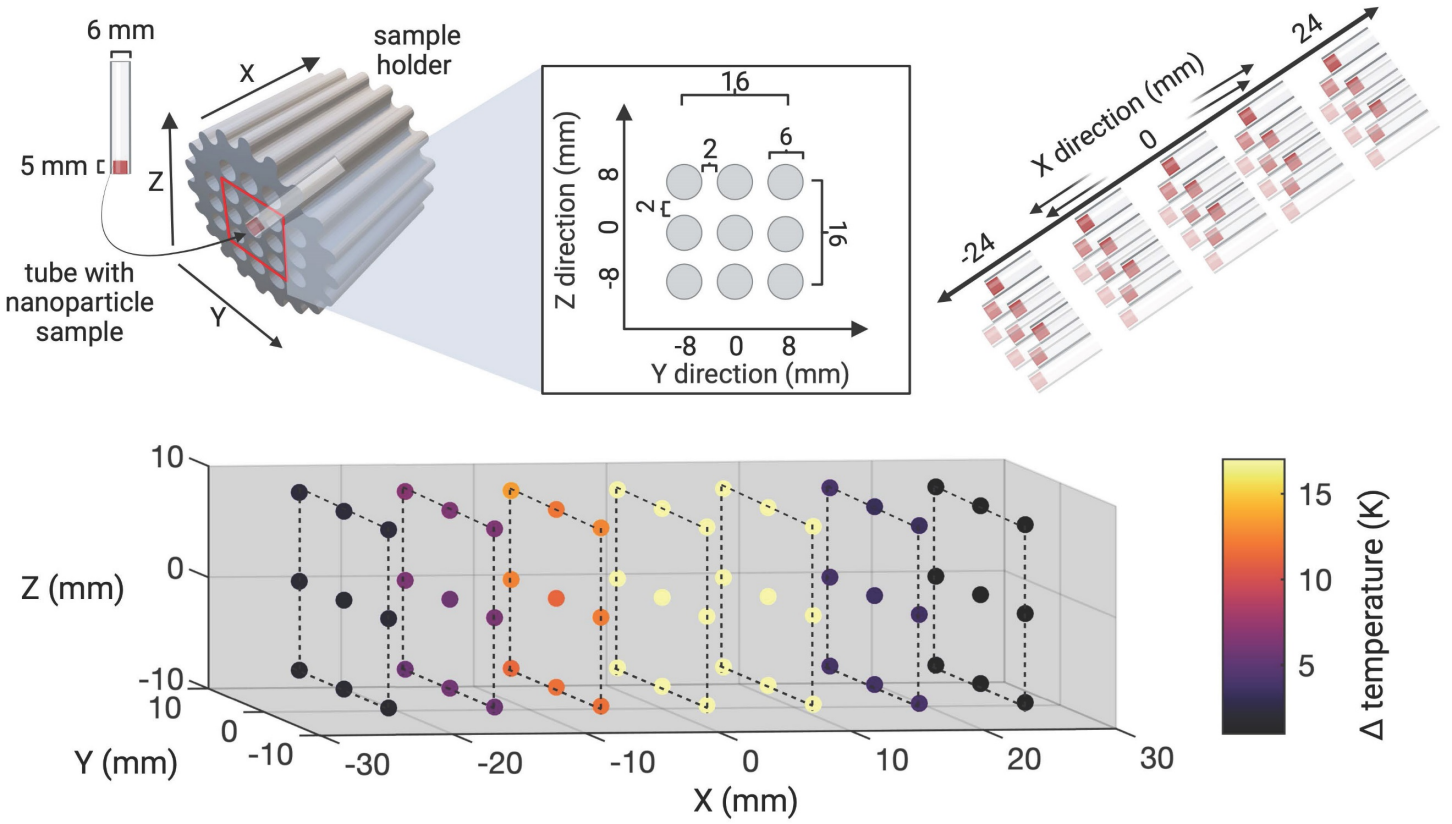
**Characterization of field of therapy**. The heating efficiency of SPION at distinct spatial locations within the MPI-MFH platform was assessed. Thin-walled glass tubes filled with 50 µl SPION (synomag-S-90, 10 mg(Fe)/mL) were placed horizontally inside a 3D-printed sample holder and distributed equidistantly throughout the cross section. The bottom of the samples was facing towards a thermal camera at approximately 1.5 m distance. With this arrangement, an area of 16 x 16 mm in the Y and Z directions at the relative center of the MPI-MFH platform (X = 0) was covered. Subsequently, MFH was applied (selection field gradients OFF, i.e., global MFH). By moving the sample holder in increments of 8 mm along the X- plane (both in relative -X and X direction), the extent of heating in the X, Y, Z directions was determined.

Subsequently, the extent of the field of therapy (FOT), i.e., the general working space for MFH and MPI, was determined by subjecting aqueous SPION samples distributed within the MPI-MFH platform to an AMF without shifting the field center through additional focus fields (selection field OFF). In the relative center of the MPI-MFH platform temperature increases of up to ΔT = 22.6 K were observed with a steady decline along the system's X-plane. Eventually, AMF became ineffective at a distance of 8 mm from the system's center in both directions of the X-plane. We conclude that the implemented MPI-MFH platform offers a usable FOT of approximately 16 mm x 16 mm x 16 mm (X, Y, Z) while using global heating with this hyperthermia insert (see Figure 2) which is in accordance with the calculated MFH field of the hyperthermia insert (see Figure 1B).

### MPI-MFH theranostic platform offers a localized field of therapy

**Figure 3:**
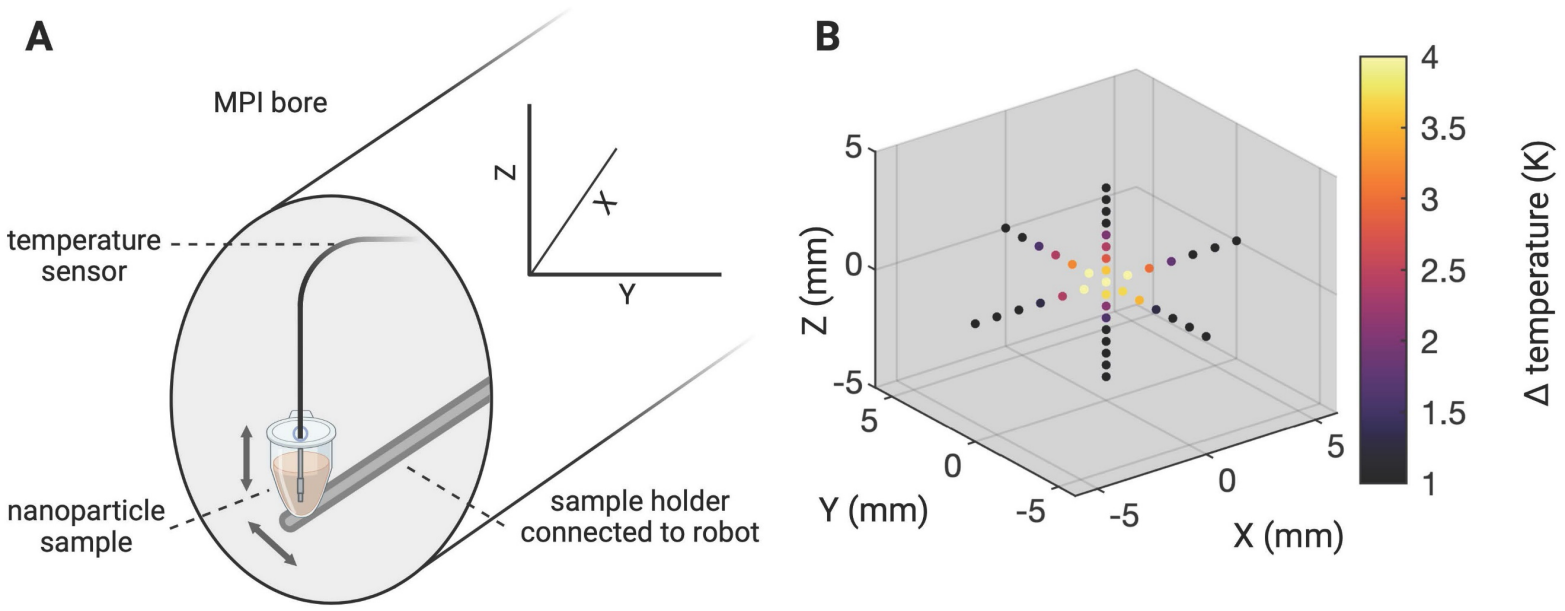
**Determining the MFH-FFR**. The MPI system's field free region (FFR) was coordinate fixed to the relative center of the MPI-MFH platform. A SPION sample (synomag-D-70, 10 mg(Fe)/mL) was subsequently moved along all three axes in small increments (1 mm in X and Y and 0.5 mm in Z direction) and subjected to AMF (300 W) revealing the MFH's point spread function relative to the FFR.

In order to minimize off-target side effects a localized application of hyperthermia is highly desired in most imaginable applications. Estimating the extent of the MFH target therefore provides crucial information on the minimal achievable therapeutic volume within our set up. With the selection field ON and FFR fixed, the highest temperature (+4.6 K) was measured at the coordinate center and predictably decreased with increasing distance along each axis (see Figure 3). Corresponding to the greater selection field gradient strength in the Z-direction, a steeper temperature distribution is observed in Z- as compared to the Y- direction (see Figure 3B). In the X- and Y- directions, the temperature increase becomes negligible beyond a distance of 5 mm from the FFR-origin (ΔT = 0 K & 0.7 K (+X & -X), and ΔT = 0.5 & 0.6 K (+Y & -Y)). In the Z- direction, no significant temperature increase was observed beyond 3 mm from the FFR (ΔT = 0.5 K and 0.6 K for +Z and -Z respectively). We thus assess the heating function of our system's fixed FFR to affect a minimal volume of 5 mm x 5 mm x 3 mm (X, Y, Z) corresponding to the selection field profile within the hyperthermia insert.

### MFH volume depends on the selection field strength and properties of the SPION solvent

**Figure 4:**
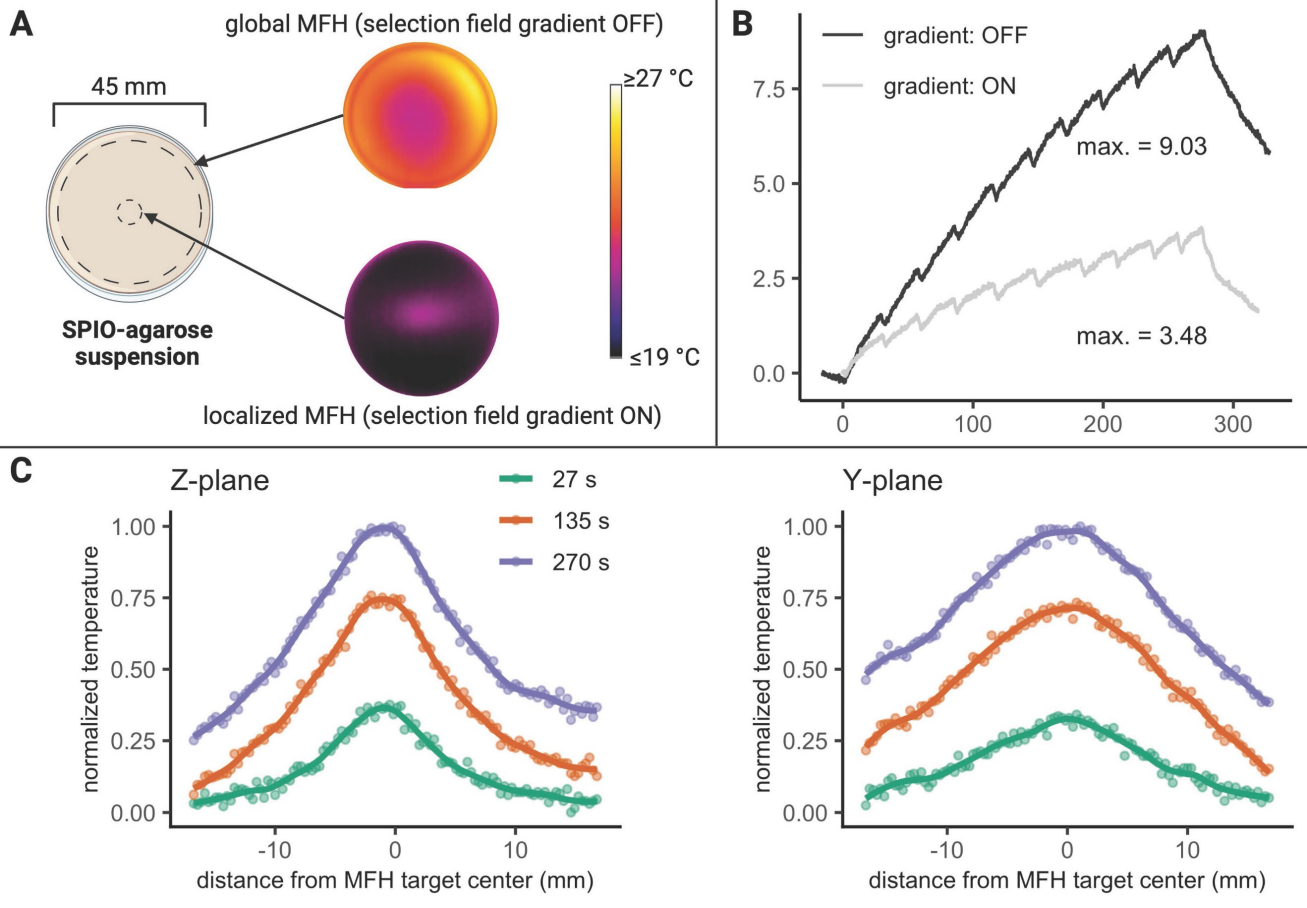
**MFH in agarose. (A)** Experimental overview of thermal camera- monitored temperature changes in a SPION-filled petri dish subjected to localized and global MFH. The MFH target during localized MFH was defined at the relative center of the MPI-MFH platform. **(B)** The corresponding temperature curves (local and global MFH) are shown versus time (the term “gradient” refers to the selection field gradient of the MPI system). **(C)** Due to the differences in selection field strengths along the three orthogonal planes within the MPI-MFH platform, the spatial extent of heating differs for the Y- and Z- axes during localized MFH. The temperature profiles at exemplary timepoints (27 s, 135 s, 270 s) during localized MFH are shown.

The actual temperature distribution inside a physical target is not only defined by the shape and size of the above estimated affected volume, but by the heat conduction properties of the target as well. The temperature distribution will become more diffuse due to intrinsic heat conduction, extending beyond the discrete points observed in air (see Figure 3). To illustrate this effect, the surface temperatures of a thin slice of agarose (0.6% w/w) containing 1 mg(Fe)/mL SPION were recorded upon localized (selection field ON) and global (selection field OFF) MFH (see Figure 4).

Prolonged global MFH application (270 s) resulted in higher maximal temperatures at the center of the sample compared to local heating of the sample center (see Figure 4B) where heat is constantly dissipating to the cooler, unheated surroundings.

During localized MFH of the sample center, substantial temperature increase in the Y- direction can be detected beyond a distance of 25 mm from the center of MFH application (see Figure 4C). In the Z- direction, the heated area extends to approximately 10 mm in both directions from the center (see Figure 4C). Assuming a similar extent of heating in the X- and Y- directions, the affected region exceeds 50 mm x 50 mm x 20 mm in hydrogel. The heating function in a continuous, heat-conducting sample is thus 10 times more blurred than the actual heat generation area determined by the FFR.

### Iron core size as predictor for heating efficiency in commercially available SPION

**Figure 5:**
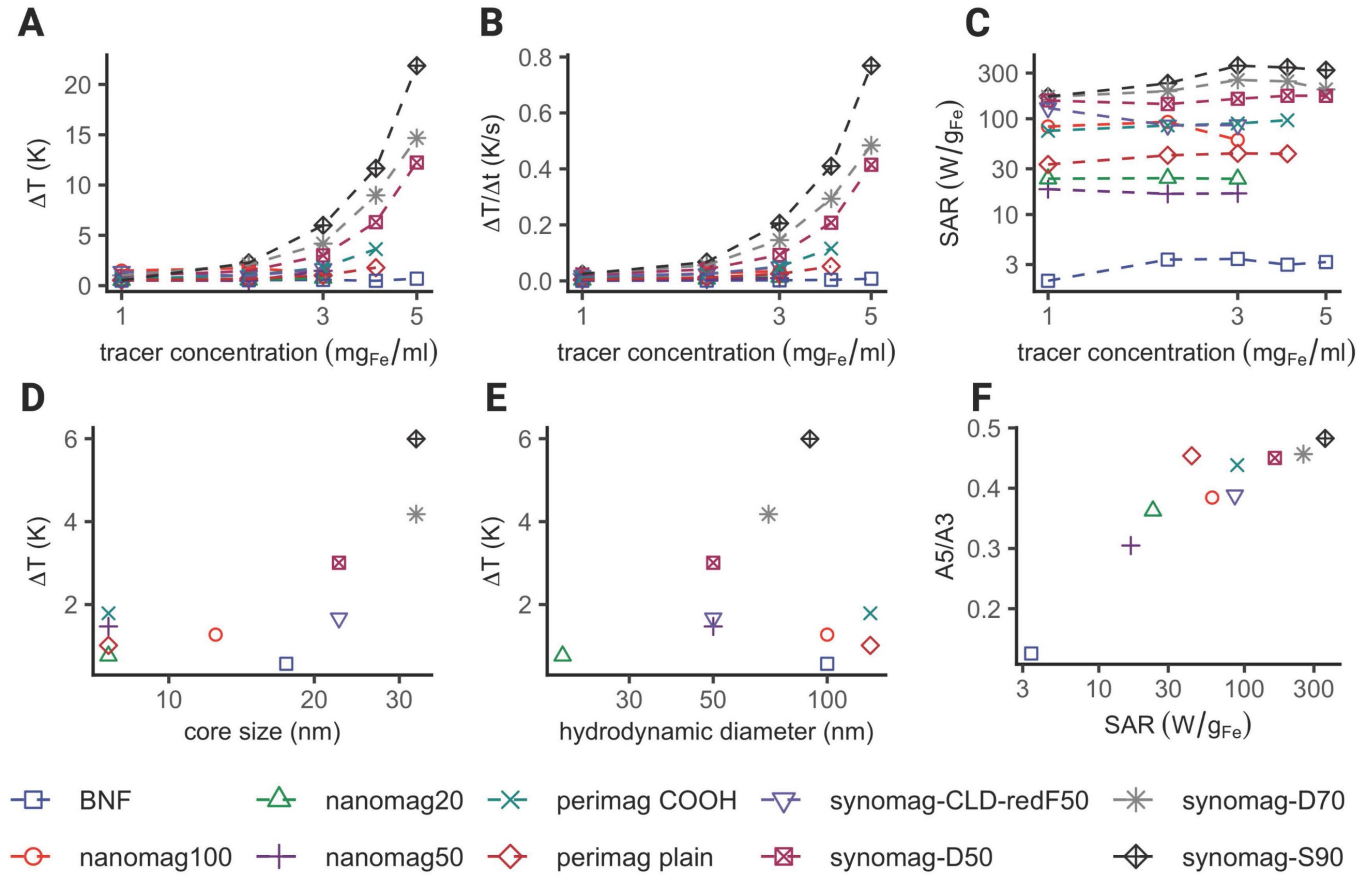
**Comparing heating efficiency of different SPION.** Display of maximally achieved temperature increase **(A)**, heating rates **(B)** and SAR **(C)** for different SPION and iron concentrations during MFH application. Dependence of core size **(D)** and hydrodynamic diameter **(E)** on maximally achieved temperature increase. **(F)** Dependence of SAR on the iron weighed magnetic moment amplitude ratio of the fifth and third harmonics (A5/A3). The A5/A3 ratios were obtained by magnetic particle spectroscopy (MPS). Measurements shown in **(D)**, **(E)** and **(F)** were performed at the same iron concentration (2.4 mg(Fe)/mL) for every SPION type.

The consideration of both image (diagnostic) quality and therapeutic efficacy (such as heating efficiency) is essential in a theranostic application. Therefore, we investigated the heating performance of 10 different commercially available SPION used for MRI, MPI and in MFH experiments. The characteristics of the SPION are summarized in Table 1. Only three of the SPION used in this study showed clear temperature increase upon MFH application (see Figure 5). As expected, the maximal temperature showed a strong dependence on the iron concentration with an overall maximal temperature increase for each SPION being achieved at their respective commercial stock concentration (see Figure 5A). Temperature increases of 37.2 K, 27.2 K and 20.6 K were measured for synomag-S-90, synomag-D-70 and synomag-D-50 respectively with correspondingly high commercial stock concentrations (10 mg(Fe)/mL). The respective SPION's rate of heating corroborates the dependency on the iron concentration (see Figure 5B) and underlines the need for MPI-based concentration mapping to optimize efficient application.

The SPION's calculated SAR in aqueous suspension showed an initial increase with increasing iron concentration for synomag-D-70, synomag-S-90 and BNF, perimag plain, and perimag COOH (see Figure 5C). This was followed by plateauing or even slightly decreasing SAR values at higher iron concentrations suggesting particle interactions. The remaining SPION samples displayed quite constant SAR values despite increasing iron concentration.

Comparing the maximal temperature achieved during MFH with the iron core size of SPION reveals a clear dependence of heating efficiency on core size (see Figure 5D). The highest temperature was achieved with the largest core size at the highest iron concentration (synomag-S-90 at 10 mg(Fe)/mL).

The maximal temperature achieved during MFH exhibits a similar dependency on the SPION hydrodynamic diameter, albeit with a reduced effect (see Figure 5E). The SPION samples with the largest hydrodynamic diameter (perimag plain and perimag COOH, Ø_hydro_ = 130 nm) showed only minimal temperature increase at stock concentration. However, when excluding SPION types that generally exhibited poor MFH performance, the dependency becomes more pronounced.

As mentioned above, theranostic SPION ideally possess both good imaging as well as MFH capability. The magnetic properties of SPION, which substantially influence their imaging quality, can be quantified by magnetic particle spectroscopy (MPS). The slope of the MPS spectrum can be parametrized by the ratio of the fifth (A5) and third (A3) harmonic amplitudes. The A5/A3 ratio has been described as an indicator for the MPI imaging resolution of SPION 31,32. SPION exhibiting a high A5/A3 ratio are considered more likely suitable for MPI. Plotting the A5/A3 ratio against the SAR, reveals that SPION exhibiting high SAR generally also show high A5/A3 values (see Figure 5F). Based on this plot, the SPIONs can broadly be ranked in terms of their theranostic performance entailing both good imaging and MFH properties. The iron-weighted magnetic moments of the samples are shown in the supplementary materials (see Figure S3). The MPS spectrum of the different SPION showed a similar course compared to the maximally achieved temperature increase, slope and SARs of the samples. For our set-up, the lowest magnetic moments and steepest decay with increasing harmonics was observed for BNF SPION. The highest magnetic moments and highest temperature increases were observed for synomag SPION.

### MPI-MFH based theranostic platform offers spatial and temporal control of hyperthermia

**Figure 6:**
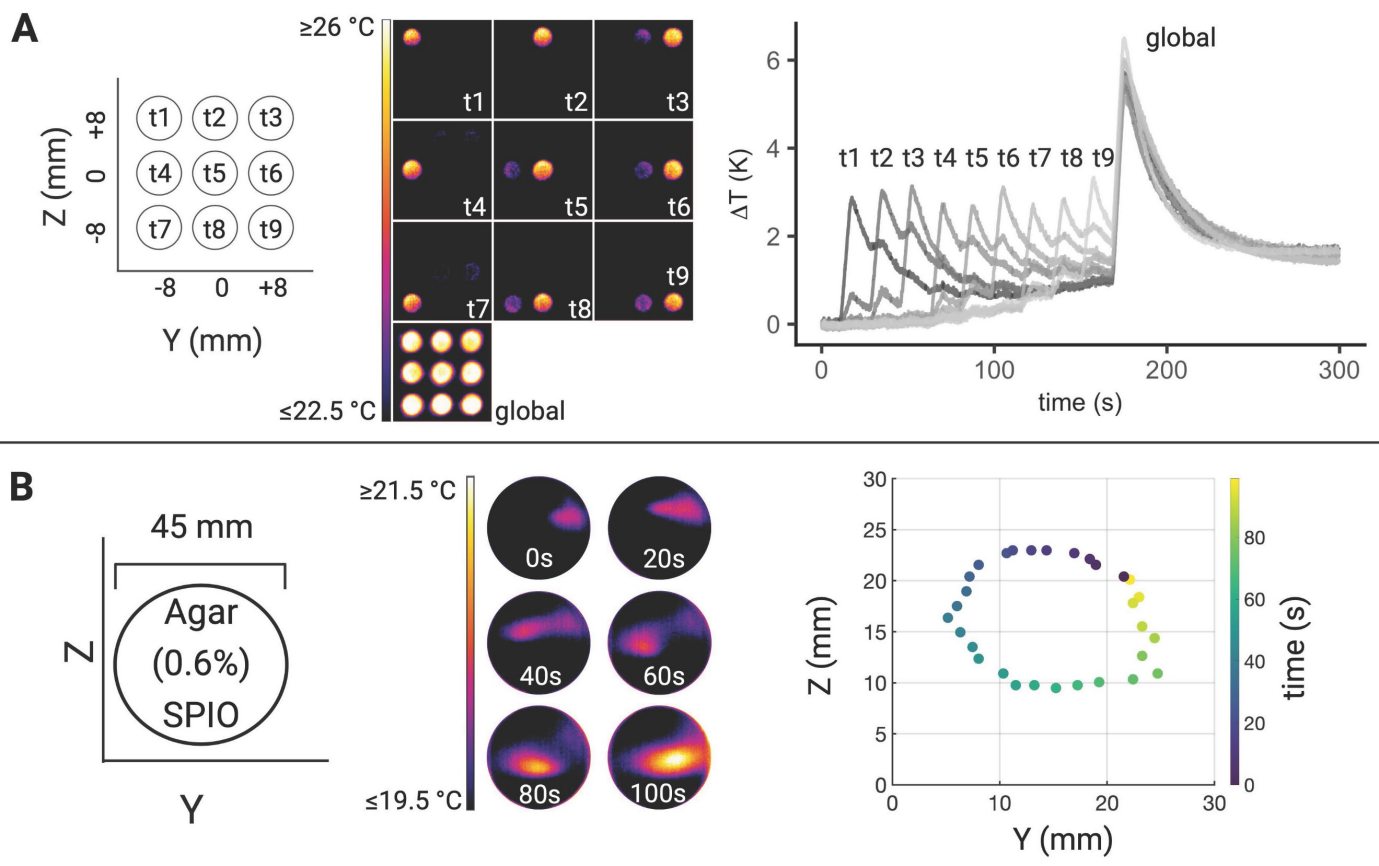
**Illustration of MFH target control. (A)** Sequence of thermal camera snapshots demonstrating temporal and spatial control of MFH targeting via field free region (FFR) shifts (left). SPION samples were positioned horizontally at defined positions within the MPI-MFH platform representing distinct MFH targets. The samples were heated successively starting with the target located in the upper left corner (relative position = 0, -8, 8 mm for X, Y, Z) and ending with global heating of all samples within the FOT (selection field gradient: OFF). The individual temperature curves of each sample are shown on the right. **(B)** MFH targeting in a continuous agarose-SPION suspension. The MFH targets describe a circle through the agarose-SPION hydrogel as displayed by the thermal camera snapshots at different representative time-points on the left. Spatio-temporal distribution of the maximal temperature on the agarose surface every 3.3 s corroborates the ellipsoid MFH trajectory (right).

In order to avoid systemic and/or off-target side effects, spatial control and local confinement of heat application during hyperthermia treatment is highly desired. By adjusting the focus field amplitudes in the X-, Y- and Z- directions, samples at different and disjoint positions can be targeted within one continuous MFH sequence. Several SPION samples were placed at different positions in the YZ- plane (X = 0) and by adjusting the corresponding focus fields, arbitrary spatial selection of MFH was demonstrated (see Figure 6). Thermal camera images show snapshots of temperature maps at different time points for independent MFH targets (see Figure 6A). The adjacent plot indicates the extent of heating relative to the base temperature prior to MFH onset for each target sample. The results display a sequential MFH-walk through each sample position, ending with global MFH application (i.e., every sample is subjected to MFH, selection field gradients: OFF).

The same approach has been applied to a homogeneous agarose-SPION mixture placed in a petri dish such that the MFH target described a circular trajectory in the SPION hydrogel (see Figure 6B). Next to the corresponding thermal camera images at selected time points, the local temperature maxima within 3.3 s intervals are plotted which describes the MFH trajectory through the agarose sample.

### MPI-MFH theranostic platform enables tomographic imaging, localized hyperthermia and multicolor thermometry

**Figure 7:**
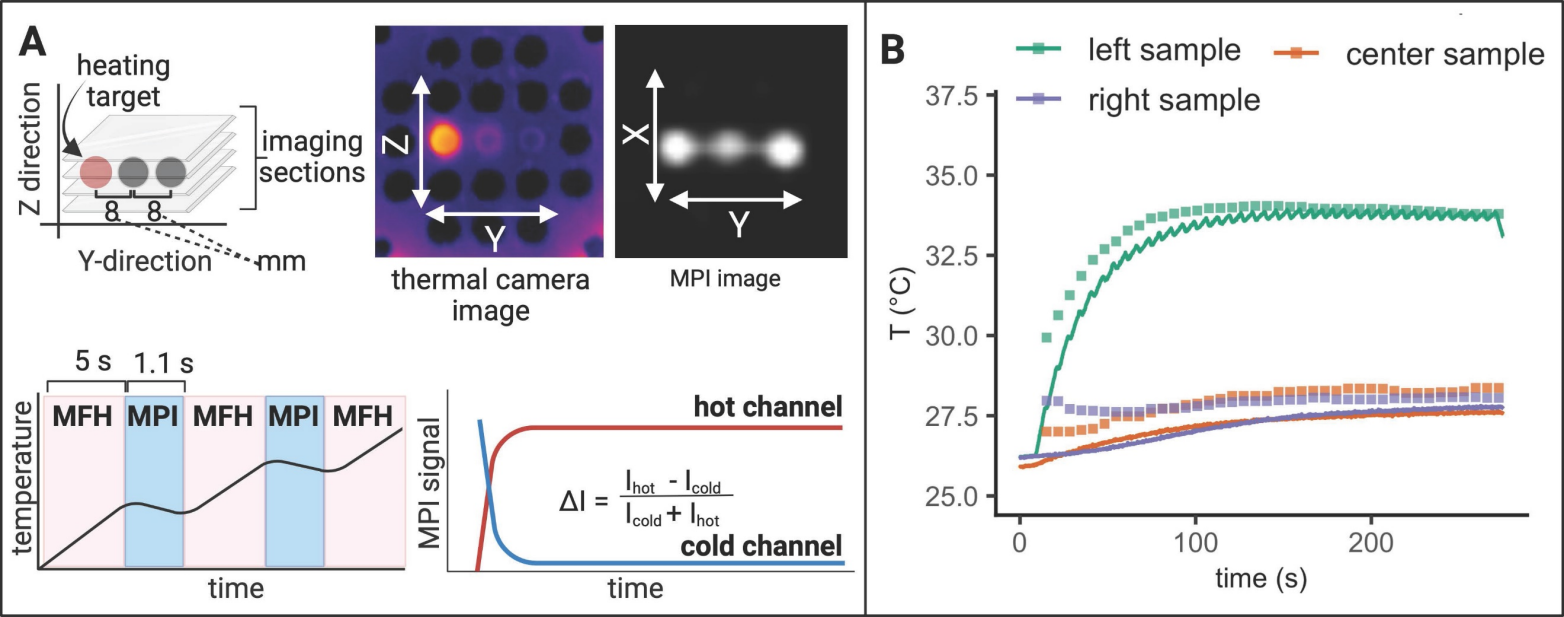
**Interleaved MPI-MFH. (A)** Three SPION samples at equidistant positions within the MPI-MFH platform (X, Z = 0 mm; Y = -8, 0, 8 mm from center) were subjected to alternating MPI-MFH sequences. By adjusting the field free region (FFR), the SPION sample positioned at Y = -8 mm, was targeted by MFH. Following each MFH application, MPI images were acquired of all samples. A sketch of the MPI-MFH sequence is depicted on the lower left. The MPI images were reconstructed applying the multi-contrast method for MPI-based thermometry (lower right). **(B)** Multi-contrast reconstructed temperature values (green, orange and purple squares) are overlaid with thermal camera measurements (green, orange and purple lines; sampling rate = 10 Hz).

To demonstrate the successful integration of a true theranostic device, consisting of an imaging and an interventional module, a sequence of alternating MPI and MFH episodes was applied to several SPION samples similar to the experimental setup used above. Additionally, the multi-contrast method for MPI-based thermometry 20 was used to retrace the sample temperatures using temperature encoded image reconstruction of the MPI images acquired during the MPI-MFH experiment (see Figure 7).

Only the left-hand SPION sample (Y = -8 mm) was subjected to MFH and consequently shows a steep temperature increase upon application onset. The temperature of the 2 adjacent samples remained relatively constant as compared to baseline. The MPI-based temperature values of the SPION samples were validated by comparing them to the thermal camera recordings (see Figure 7B). The temperature increase was both observed by thermal camera recordings as well as with MPI-based results.

Since every MPI-MFH cycle presented here starts with MFH application (see Figure 7A bottom left), the first MPI- based temperature value is acquired just after the first MFH block of the cycle. For every MPI-MFH cycle, MFH was applied for a duration of 5 s and image acquisition (MPI) was done over a duration of 1.1 s. Consequently, MPI- based temperature values were acquired every 6.6 s.

The largest difference between the two modes of temperature measurement was observed at MFH-MPI onset. For the heated sample (Y = -8 mm), thermal camera measurements exhibited a value of 27.8 °C while the MPI-based reconstructed temperature was determined to be 29.9 °C. During the initial temperature increase, the MPI reconstructed temperature values progressively approached the values measured by the thermal camera, ultimately aligning with them approximately 140 seconds after MPI-MFH onset. Therefore, the two modalities exhibit a comparable temperature value following the last MFH application, with thermal camera recordings indicating 33.8 °C and MPI-based temperature reconstruction indicating 33.9 °C.

Subsequent to the first MPI-MFH cycle, the central samples' (Y = 0 mm) temperature was 27.0 °C according to the MPI- based multi-contrast method while thermal camera measurements showed 26.0 °C. Both temperature measurement modalities showed a similar temperature progression where image reconstructed values maintained the initial offset with respect to the thermal camera measured temperature. The temperature values following the last MFH application were 28.4 °C (MPI reconstructed) and 27.6 °C (thermal camera measured).

Analog to the other samples, in the right-hand sample (Y = 8 mm), reconstructed (28.0 °C) and thermal camera recorded (26.2 °C) temperature values showed a slight disparity following the initial MFH application. The subsequent temperature profile depicts a similar progression between both reconstructed and measured values. Following the last MFH application, 28.1 °C and 27.7 °C were observed (MPI reconstructed and thermal camera measured respectively).

In general, the MPI-reconstructed temperature profiles of the three samples aligned with the data obtained from thermal camera measurements. The resulting reconstructed (34.0 °C, 27.7 °C and 27.9 °C for y = -8, 0, 8 mm respectively) and thermal camera measured (34.1 °C, 28.4 °C and 28.2 °C for y = -8, 0, 8 mm respectively) maximal temperature values were observed to be similar.

### MPI-MFH platform adjusts to new hyperthermia target during application

**Figure 8:**
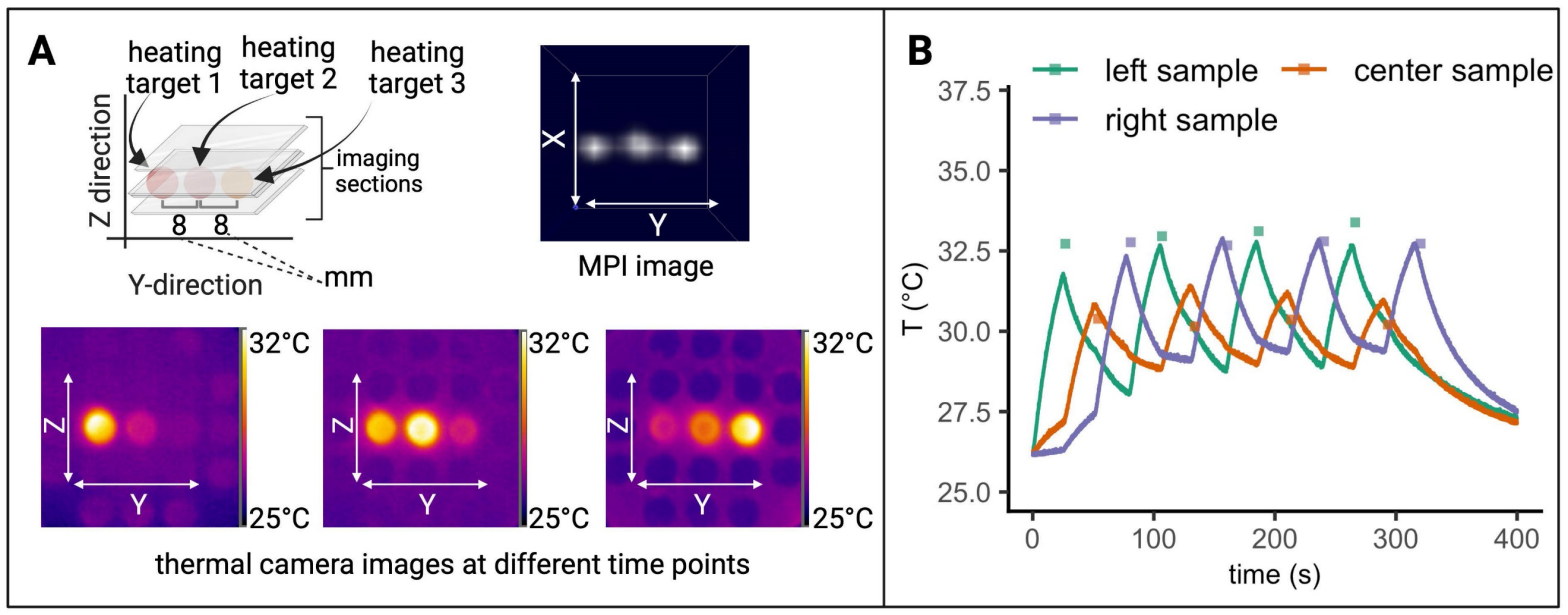
**Interleaved MFH and MPI with alternating MFH targets. (A)** Three SPION samples at equidistant positions within the MPI-MFH platform (X, Z = 0 mm; Y = -8, 0 and 8 mm from center) were subjected to alternating MPI-MFH sequences. By adjusting the MFH-FFR, the SPION samples were targeted successively while the temperature profile was monitored with a thermal camera. **(B)** Multi-color reconstructed temperature values (green, red and purple rectangles) overlaid with thermal camera measurements (sampling rate of approximately 10Hz (green, red and purple lines)).

The previous two sections showed the feasibility of MFH-target localization and interleaving MPI-MFH sequences with subsequent MPI based thermometry, respectively. In the following, we present the results of the combination of both, i.e., an MPI-MFH platform with arbitrarily alterable theranostic targets (see Figure 8).

Analog to the experimental set up described above, three samples were targeted with interleaved MPI-MFH cycles. MPI images were acquired using a FOV that covered all three samples. Consequently, with every MPI-MFH cycle an MPI- based temperature value for each sample was obtained. The MFH target however, was changed periodically at the start of every MPI-MFH cycle. The thermal camera measurements (see Figure 8B, red, purple & green lines), as well as MPI reconstructed temperature values (see Figure 8B, red, purple & green squares), show the successive heating of the SPION samples beginning with the left-hand sample. The MPI images acquired during MPI-MFH sequences (see Figure 8A upper right) served as basis for subsequent MPI-based multi-contrast thermometry. Analog to the above-described results for a fixed MFH target, the MPI reconstructed temperature values are similar to the thermal camera recordings.

### MPI-MFH procedure is limited in circulatory system

This study aims to verify the general characteristics of an MPI and MFH-based theranostic platform. As most reports on treatment with MFH utilized accumulated or injected SPION 29,33, stationary phantoms with well-defined SPION samples seemed to be adequate test objects. However, systemic SPION application (e.g., by i.v. injection) is not well represented by static phantoms and the gap to *in vivo* applications has yet to be overcome. In an attempt to at least narrow it, we included one crucial parameter into our phantoms: The effect of forced transport of SPION solutions through the MFH-target volume (i.e., phantom blood flow circulation) on the MFH performance.

**Figure 9:**
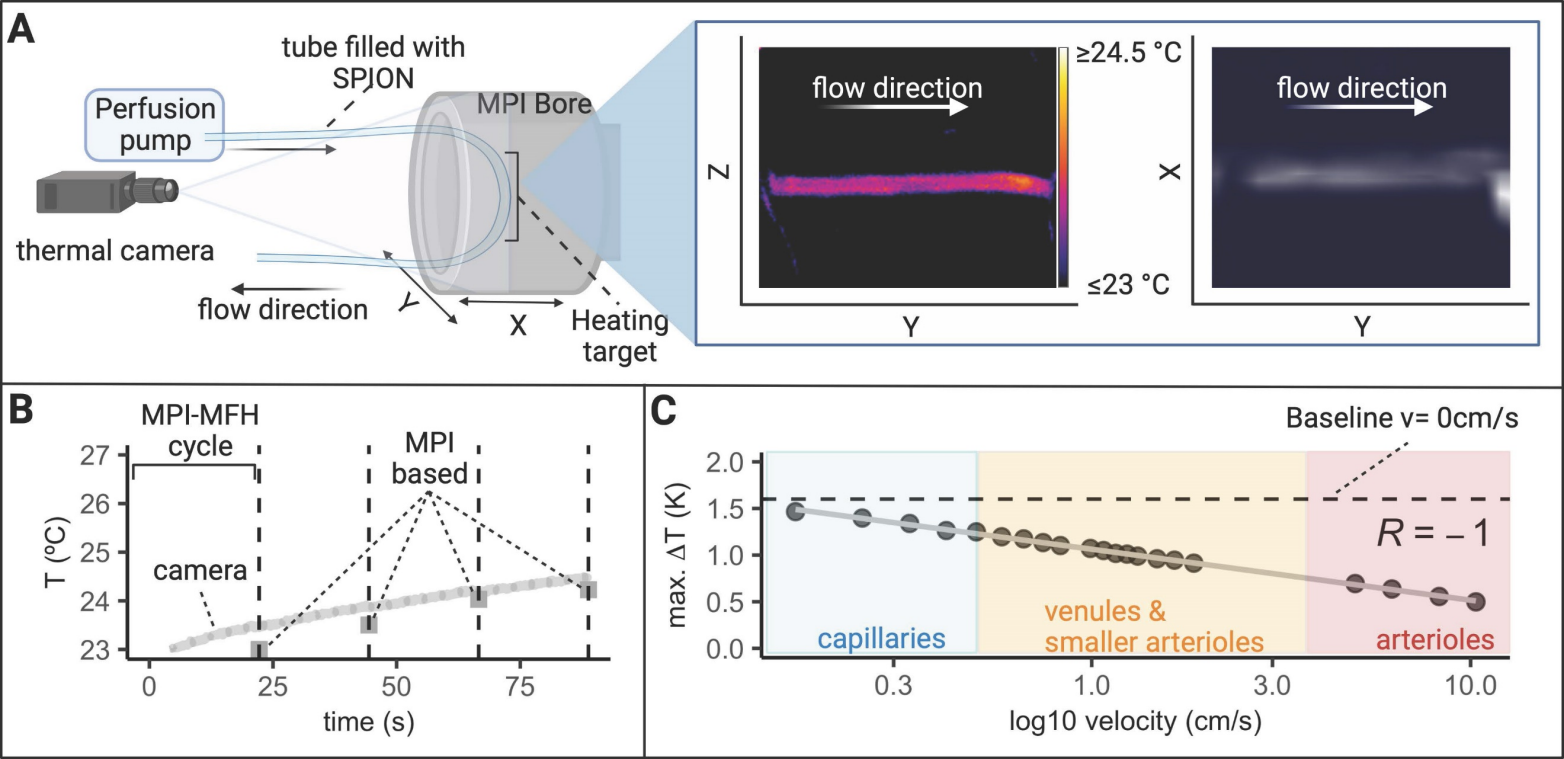
**Effect of MFH in a flow phantom. (A)** A flow system connected to a tube filled with SPION was placed inside the MPI bore to mimic (blood) circulation and investigate the effect of circulation velocity on heating efficiency. A thermal camera was placed in front of the MPI bore such that the heating target (inside the AMF of the hyperthermia insert) as well as portions of the circulation tube outside the AMF were visible. The circulation phantom was targeted with interleaved MPI and MFH cycles successively heating and imaging the circulating SPION. A representative thermal camera image during MPI-MFH application (at a flow velocity of 0.4 ml/min; 0.33 cm/s) and the corresponding MPI image are shown. **(B)** Each MPI-MFH session consisted of 4 cycles with a duration of 24 s each. Acquired MPI images were used to reconstruct temperature progression during MFH using the MPI-based multicolor method (4 separate grey rectangles) and plotted against the thermal camera measurements (light grey curve). **(C)** The maximal temperature of 4 successive MPI-MFH cycles were plotted for different flow velocities. Flow velocities of different human blood vessel types 39 were approximated with the results and a linear regression model was fitted to the data points.

A circulatory tubing system in plain sight of the thermal camera was placed within the bore of our MPI-MFH platform (see Figure 9). The tube was connected to a perfusion system outside the MPI which allowed for incremental adjustment of the SPION velocity within the tube. The entire visible length of the tube was subjected to cycles of interleaved MPI-MFH at various representative circulation velocities. Without flow (v_0_ = 0 cm/s), a maximal temperature increase of 1.6 K was observed, while at the maximal velocity (v= 10.33 cm/s), the temperature increase was 0.5 K. An example MPI image of the circulation tube (v = 0.33 cm/s) and the corresponding thermal camera image following the last MPI-MFH cycle is depicted in Figure 9A on the right. MPI-based multi-contrast reconstruction of the temperature profile during MPI-MFH application was applied for each of the indicated flow velocities. However, only reconstruction of the slowest velocities (0, 0.17, 0.25, 0.33, 0.41 cm/s) showed similar values to the corresponding temperature readings from the thermal camera. An exemplary temperature progression (v = 0 cm/s) in combination with the multi-contrast reconstructed temperature values are shown in Figure 9B.

## Materials and Methods

### Temperature measurements

During experiments, if not specifically stated otherwise, temperature profiles were recorded using a thermal camera (FLIR Systems, A8303sc with custom macro lens, 20 mK temperature resolution and 30 Hz frame rate and 308 µm pixel size) that was placed approximately 1.5 m in front of the MPI scanner system with unobstructed line of sight inside the bore. Temperature progressions were analyzed by manually defining ROIs covering the respective sample and extracting the maximal temperature per time point. Specific emissivity of sample tube material was not taken into account.

### MFH applications in SPION samples

If not stated otherwise, all temperature measurements during MFH application were done in a 3D printed circular sample holder (Ø59 mm, L = 48 mm) designed to fit the hyperthermia insert and equipped with several notches to allow for sufficient airflow (see Figure 1C). The sample holder can accommodate 9 samples at once, spaced 2 mm apart in the Y- and Z- directions (8 mm from center to center). Thin-walled glass tubes (6 mm x 50 mm, Disposable culture tubes, Borosilicate Glass, Kimble) served as punctiform MFH targets. The glass tubes were sealed off with paraffin to avoid drying out. The glass tubes were placed horizontally inside the sample holder, the bottom facing the thermal camera such that a circular target is presented, resulting in a 3 x 3 grid spanning the Y- and Z- directions between -8 and +8 mm relative to the hyperthermia insert's center for any given location on the X plane.

As proof of concept, the sample tubes filled with 140 µl of SPION suspension (synomag-S-90, 10 mg(Fe)/mL) or 140 µl of water, were heated in 2 separate MFH sessions using the maximal power of 600 W with 2 heating cycles of 22 s each.

### Characterization of field of therapy

Upon equipping a sample holder (see Figure 1C) with SPION samples positioned at all locations in the Y- and Z- directions, the entire sample holder was moved by means of a 3-axis robot through the hyperthermia insert in the X- direction in seven 8 mm increments going from -24 mm to +24 mm relative to the hyperthermia insert's center. At each incremental position, MFH was applied to the entire inner volume of the hyperthermia insert (global MFH) yielding a total of 63 individual positions and covering an area of 48 mm x 16 mm x 16 mm (X, Y, Z). A sketch of the experimental set up is depicted in Figure 2. MFH was applied using the maximal power of the hyperthermia insert (600 W) with two heating cycles of 25 s each.

### 3D Characterization of localized FFR-MFH

To precisely determine the system's heat production function, i.e., the extent of SPION heating along the orthogonal planes of the MPI-MFH experimental set up, the location of the FFR was fixed at the center of the hyperthermia insert. Localized MFH was achieved by selection field gradients in the X, Y and Z- directions of 1.25 T/m, 1.25 T/m and 2.5 T/m respectively. A punctiform calibration sample was attached to a rod and moved by means of a 3-axis robot to each position inside the MPI-MFH platform's FOT in 1 mm (in X- and Y- direction) and 0.5 mm (in Z- direction) increments (see Figure 3 left), analog to the process used for system matrix acquisition. The calibration sample consisted of a temperature-stable PVC tube filled with 27 µl SPION suspension (synomag-D-70, plain, micromod Germany, 10 mg(Fe)/mL). MFH was applied (P = 300 W, 1 cycle of 30 s) at each spatial location. The temperature of the SPION sample was monitored by a commercial fiber optic temperature sensor (TS2, Weidmann-Optocon, Germany) introduced through the lid of the PVC tube.

### Characterization of localized and global MFH in a continuous hydrogel sample

A SPION (synomag-D-70) suspension was added to molten agarose (0.6% by weight; simulating brain tissue viscoelasticity 34) leading to a concentration of 1 mg(Fe)/mL in a culture dish (glass bottom dish 50 mm x 7 mm, Wilco Wells). Upon solidifying to an approximately 5 mm thick layer, the dish was placed upright in the center of the hyperthermia insert, such that the circular surface of uncovered agarose faced a thermal camera for thermal monitoring. Thereby, heating of the thin agarose-layer could be observed in the Y- and Z- directions on the surface of the hydrogel. The SPION-agarose suspension target was heated (P = 600 W) either locally (selection field gradients: ON; 1 T/m, 1 T/m, 2 T/m (X, Y, Z) with the FFR located at the center of the MPI-MFH system or globally (selection field OFF). An overview of the experimental set up is shown in Figure 4A.

For comparison of the maximally achieved temperature during both measurements (selection field gradients ON vs. OFF), a circular ROI was manually defined within the software of the thermal camera (FLIR Research Studio) at the center of the culture dish, and the maximal temperature was extracted over time. The extent of heating during localized MFH was determined by extracting the temperature values corresponding to the Y, Z plane of the MPI-MFH platform along 2 orthogonal axes through the center of the monitored agarose suspension (see Figure 4B).

### Targeting the field of therapy

The selection field gradients define the spatial resolution during MPI 8 by influencing the extent of the FFR. Simultaneously, the magnetization response of the deployed SPION influences the spatial resolution: A steeper magnetization slope leads to higher spatial selectivity 35.

A similar mechanism is expected to apply to MPI-based MFH targeting. Component parameters of the MPI system define the extent of the FOT during AMF applications and the point spread function of the FFR defines the focus of the MFH treatment since only SPION within the FFR are susceptible to time-varying changes. Finally, any displacement of the FFR using focus fields allows for a corresponding shift of MFH target to any desired spatial location within the MFH FOT.

The above-described sample holder (see Figure 1C) was used to demonstrate directional control of MFH application. The phantom was equipped with 9 SPION samples (synomag-S-90, 5 mg(Fe)/mL, micromod Germany, 5mg (Fe)/mL) and placed at the center of the hyperthermia insert. Each of the sample locations (in Y- and Z- direction) was targeted successively (P = 600 W, 1 MFH cycle of 10 s). MFH localization was achieved by defining a selection field gradient of 1 T/m, 1 T/m, 2 T/m, (X, Y, Z).

### Comparison of different SPION

In order to optimize theranostic applications, ideal SPION should exhibit good imaging capabilities along with good MFH properties. In this study, we conducted a comparative analysis of various commercially available SPION to assess their heat-generating capacity using the introduced setup. The characteristics of the different SPION types are listed in Table 1. SPION types were used that have shown good MPI and MRI quality (perimag (-plain (dextran coating); -COOH (dextran coating with attached carboxyl group)), nanomag-D (dextran coating)) 15,36, high SAR when subjected to AMFs (BNF-Dextran) 37 or both (synomag (-D (dextran coating); -S (starch coating); -CLD-redF (dextran coated and fluorescently labeled)) 15,36,38.

The described SPION sample holder (see Figure 1C) was used for comparison of the MFH performance within the MPI-MFH platform in successive separate MFH sessions at the same specifically chosen spatial locations to account for any potential phantom or gradient related inhomogeneity. Different SPION sample concentrations (0.6, 1.2, 2.4 mg(Fe)/mL were compared across all SPION types, and 5 and 10 mg(Fe)/mL for SPION with correspondingly high stock concentrations). The individual specific commercial stock concentration served as upper concentration limits for the respective SPION (see Table 1).

MFH (P = 600 W) was applied for two consecutive heating cycles with a duration of 20 s each. The SAR of each sample was then calculated from the temperature profiles using following formula:


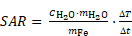

(1)

Where 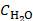 is the heat capacity of water (4.168 J/gK), 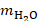 and 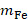 are the masses of water and iron in the sample respectively, and 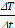 is the slope of temperature increase during the first 20 s of MFH application.

MPS measurements (pure devices, drive field = 25 mT, excitation frequency = 25 kHz) were performed using 50 µl of each SPION sample (Table 1) at their respective stock concentration. For comparison, the results were normalized for the different iron concentrations.

### Interleaved MPI-MFH combined with MPI thermometry

The above-described sample holder (see Figure 1C) equipped with three SPION samples was placed inside the MPI-MFH system such that the center of each of the samples was located at X, Z = 0. In the Y- direction, the samples were positioned at +8 mm, 0 mm & -8 mm (see Figure 1A) relative to the MPI-MFH platform's center.

Periodic image acquisition (MPI) of the 3 samples was alternated with the MFH sequences, targeting the SPION sample at Y = -8 mm (see Figure 5). MPI-MFH sessions consisted of 40 individual cycles with a duration of 6.1 s each (1.1 s of MPI and 5 s of MFH), corresponding to a total duration of 248 s. MPI-MFH cycles were applied using the following parameters: bandwidth: 1.25 MHz, drive field strength: 14, 14, 14 mT (X, Y, Z), selection field gradient strength: 1, 1, 2 T/m (X, Y, Z), drive field FOV: 28, 28, 14 mm (x, y, z), FOV (analog to FOT): 40, 40, 20 mm (X, Y, Z). Images were acquired with matrix size = 20, 20, 20 (X, Y, Z) resulting in a resolution of 2, 2, 1 mm (X, Y, Z) per voxel.

The acquired images were subsequently used for MPI thermometry utilizing the multi-contrast method 20. MPI thermometry is based on the calibration of the MPI system to differently tempered SPION samples. Corresponding system matrices are acquired defining the temperature range of the SPION that can be detected within an imaging session. Here the lower base temperature of the sample (“cold channel”) and the maximal achieved temperature (“hot channel”) are chosen as lower and upper temperature boundaries. As the MPI signal and the SPION temperature is linearly dependent, temperature profiles of samples in a given imaging session can be obtained by reconstructing the MPI signals using both hot and cold channels 20.

Following the multi-contrast method, two system matrices were acquired at 45 °C (hot channel) and 23 °C (cold channel). For that, a water bath tempered SPION sample (synomag-S-90, micromod Germany, 5 mg(Fe)/mL) was mounted on the computer-controlled sample rod used for system matrix acquisition in our setup.

MPI images were reconstructed with the two system matrices representing base and maximal temperature using the following reconstruction parameters: relative regularization value: 0.01, number of iterations: 15, SNR threshold: 10 and border frequency: 0.625 MHz. The spatial resolution of the temperature maps originates directly from the imaging parameters.

### Interleaved MPI-MFH of alternating MFH targets combined with MPI thermometry

In an experimental set up analog to the one described in the previous section, the objective was expanded to include alternating MFH targets during sequences of MPI-MFH application. Starting with the SPION sample located at the relative position Y = -8 mm, the samples were targeted in succession while images of all three samples were acquired for the duration of the measurement (see Figure 8).

Here, MPI-MFH sessions consisted of 4 individual cycles. One MPI-MFH cycle is completed, as soon as all of the 3 samples were targeted once (i.e., one cycle contained 3 MFH and 3 MPI sessions; one for each sample respectively). Each MPI-MFH cycle had a duration of 79 s (3 x 24 s of MFH and 3 x 2 s of MPI) corresponding to a total duration of 320 s. Reconstruction of MPI-based temperature values was accomplished using the same system matrices and reconstruction parameters as described in the previous section.

### Variations of flow velocity during MPI-MFH

To study the effect of flow velocity on heating efficiency within the MPI-MFH platform, 10 ml of a SPION (synomag-D-70)- suspension at a concentration of 1 mg(Fe)/mL were loaded to a flow system driven by a perfusion pump (PPS2, Multichannel Systems, Harvard Bioscience Inc.) connected to a tubing-loop (loop length= 375 cm, Ø_inner_ = 0.2 mm, Ø_outer_ = 0.3 mm, silicone tubing, Tygon) (see Figure 9A). The tube was placed at the relative position of X, Z = 0 such that the tubing segment serving as the theranostic target covered almost the entire length of the MPI bore in Y- direction while the two ends of the tube were guided along a 3D printed holder to the perfusion device outside the MPI system. The experimental set up is depicted in Figure 9A.

An alternating sequence of MFH and MPI (4 block repetitions of 21 s MFH and 1 s MPI) was used to heat and image at selected flow velocities the horizontal segment of the tubing. The maximal temperature increase achieved during global heating (600 W, selection field gradients: OFF) was analyzed for different flow velocities and put in relation to the approximate velocities of differently sized blood vessels in humans 39.

The set up allowed for circulation of the SPION suspension in 0.1 ml/min increments. The SPION suspension was pumped at different flow rates (0, 0.2, 0.4, 0.5, 0.6, 0.7, 0.8, 0.9, 1, 1.2, 1.3, 1.4, 1.5, 1.6,1.7, 1.8, 1.9, 2.25, 6, 10, 12 ml/min) corresponding in the used set up to circulation velocities of 0, 0.25, 0.33, 0.41, 0.50, 0.58, 0.66, 0.74, 0.83, 1.00, 1.07, 1.16, 1.24, 1.32, 1.49, 1.65, 1.86, 4.96, 6.20, 8.26, 10.33 cm/s respectively.

MPI images were acquired with the following parameters: matrix size = 15, 15, 5 (X, Y, Z), FOV (analog to FOT) = 28, 28, 14 mm (X, Y, Z), drive field strength = 14, 14, 14 mT (X, Y, Z), selection field gradient strength = 1, 1, 2 T/m (X, Y, Z), drive field FOV = 28, 28, 14 mm (X, Y, Z) and bandwidth = 1.25 MHz.

MPI based multi-contrast reconstruction, following the method described above, was achieved with the “cold channel” acquired at 23 °C and the “hot channel” acquired at 27 °C which reflects the assumed temperature range during the circulation-phantom experiment. The number of the system matrix' measurement averages was set to 50 resulting in a total acquisition time of approximately 7 h 44 m for each channel.

MPI images were subsequently reconstructed with the two system matrices using the following parameters: relative regularization value: 0.01, iterations: 15, SNR threshold: 10 and border frequency: 0.625 MHz.

## Discussion & Conclusion

### MPI-MFH theranostic platforms

Theranostic platforms have gathered significant interest due to their ability to minimize invasiveness, off-target effects, and to augment real-time reactivity during application 40-42. With high imaging contrast and high sensitivity, the possibility of external control over the therapy's localization, and the dual purpose of SPION with no depth limitations in tissue, integrated MPI-MFH is an optimal candidate for theranostic applications 11,43,44. The first integrated MPI-MFH platform was introduced in 2017 45. Here, the authors describe, through simulations and phantom experiments, a back-to-back MPI-MFH platform that entails a field free line which enabled MFH target localization in 2D.

MPI-guided MFH has been demonstrated with great success regarding tumor localization and targeted MFH delivery *in vivo* 29. In this case, the MPI-MFH workflow consisted of back-to-back separate systems involving one localized MFH system to deliver the therapeutic component and one separate MPI scanner for imaging. In the study at hand, the general feasibility of an MPI based theranostic platform enabling versatile three-dimensional tomographic imaging in three dimensions, highly localized MFH as well as thermometry within the same system was demonstrated.

### Transition into clinical application

In order to facilitate the transition of the MPI-MFH platform into clinical applications, there are two major barriers that need to be addressed. The first one is related to the instrumentation: Going beyond commercially available pre-clinical MPI scanners, research on human sized systems has been introduced 46,47. Here the main challenge is to maintain high magnetic field gradients in large instruments. Further, the corresponding requirements for cooling and power expenditure are quite challenging, which to date is a major issue in whole body scanners 46,48.

The second key challenge is to maintain SPION dosages within clinical limits and simultaneously achieve local iron concentrations sufficient for adequate heating with high SAR. Existing SPION have not been optimized for heating but for MRI, leveraging their role as contrast agents to offer valuable insights into anatomical structures cleared by the reticuloendothelial system 12,49. This application has played a crucial role in the development of safe and biocompatible SPION, enabling a seamless transition for their *in vivo* application during MPI but not for optimal MFH.

In this regard, the current practice of injecting SPION into tumors for MFH treatments ensures that the systemic concentration is intentionally maintained at low levels while high concentration at the tumor site is achieved 29. Smart functionalized SPION might home in on cancer cells noninvasively 50 which opens the door for less invasive systemic application of SPION and consequently treatment of deep-seated tumors. However, MPI-MFH may not be restricted to the treatment of solid tumors. It can for example be envisioned, that i.v. injected SPION are subjected to mild MFH at the capillaries of the blood-brain-barrier for transient and localized permeability increase to enable targeted transport of pharmaceuticals into the brain. *In vivo* studies have shown no systemic toxic effects at concentrations up to 100 mg_Fe_/kg following i.v. injections of SPION 51. However, the MFH efficiency of circulating SPION will highly depend on the circulation velocity as has been exemplified by our circulation phantom. Already at comparatively low circulation velocities, i.e., in small blood vessels, heating performance is substantially decreased. Hence, drastically higher SPION concentrations are needed for MFH targeting circulating SPION. During localized MFH in a passively cooled SPION-agarose suspension, we observed an increasingly blurred “heating spot” during localized MFH application in an (see Figure 4C). This observation does not relate to any *in vivo* application, because live tissue is constantly cooled by blood perfusion.

The highest discrepancy between multi-contrast reconstructed and thermal camera monitored temperature values was observed for high velocities in the circulation phantom (see Figure 9). It should be considered, that the small temperature increases observed were achieved with very short MFH application in 10 ml tube volume as compared to the 140 µl volume of the glass vials with commercial SPION. Here motion artifacts may occur, leading to an ill-posed inverse problem resulting in a lower reliability on the thermometry. This could potentially be overcome by calibrating for the movement if it is known 52. We conclude that the limit of multi-contrast temperature reconstruction is reached at higher circulation velocities, which is confined by the comparatively slight temperature increase achieved during MFH of SPION circulating at high velocity. However, thermal camera measurements have to be considered lower bounds for SPION temperature, as the tubing's silicone wall may act as insulator obscuring the inner temperature from the outside wall. Further, the emissivity of the SPION sample vessel might have an effect on the results of the thermal camera measurements as well. As sample tubes of the same material were used for the temperature comparisons, this effect can be considered systematic. Previously, we were able to show high similarity between fiber optic thermometer measured and MPI reconstructed temperature values during laser heating of a SPION suspension within the same commercial MPI used in this manuscript 53. Therefore, we assume that thermal camera measurements, within the context of the here presented experiments, provided adequate control temperature values.

In addition to the circulation velocity and consequently low temperature increase following MFH application, the effectiveness of the multi-contrast method will suffer from various other factors during *in vivo* conditions. For example, the small blood volume and low iron concentration as well as different SPION suspension viscosities depending on the specific target location, can drastically decrease the accuracy of MPI based thermometry *in vivo*. One contributing factor is the system matrix which is typically obtained under *in vitro* conditions, and fails to capture the intricate dynamics of living organisms. To address this, efforts can be directed towards emulating *in vivo* conditions more closely during acquisitions, such as utilizing SPION-blood-tissue suspensions for calibration. Accuracy can be further improved by optimizing the physical properties of SPION regarding image quality such as their shape and size.

Generally, we observed good MFH properties in SPION, that also displayed high measured magnetic moments. This may indicate that SPION properties improving image quality also improves their MFH performance. It should be noted here, that the MFH efficiency of the commercial SPION was assessed with our specific experimental set up. Different AMF frequencies may lead to different MFH performances for individual SPION samples. In fact, BNF SPION have shown SARs of up to approximately 400 W/g_Fe_ using different AMF frequencies and magnetic field strengths 54 while with the presented system only an SAR of approximately 3 W/g_Fe_ was achieved. Tailoring the AMF parameters to the individual properties of different SPION is therefore also crucial for MFH optimization.

Other aspects of already existing SPION can be modified in order to increase SPION concentration at the target site. Quite desirably, SPIONs should be equipped with additional polymer coating to allow a longer residence time in the blood circulation 55,56 and thereby enhance MPI as well as MFH performance.

## Conclusion & Outlook

We were able to demonstrate precise spatial control at the millimeter scale for imaging and MFH application, along with satisfactory resolution for MPI- based thermometry *in situ*. Interleaving MPI and MFH sequences within one platform offers the potential for a therapeutic feedback loop by periodically imaging the targeted SPION within the FOT. Real-time monitoring of the FOT by means of MPI based thermometry would subsequently enable the adjustment and control of the MFH therapy parameters. In clinical applications, this would enable safe delivery of MFH therapy ensuring live control of a specific target temperature. Demonstrating an integrated MPI-MFH feed-back loop in the commercial control software is certainly the next step towards optimization of the theranostic application. The presented theranostic system will aid to visualize potentially functionalized SPION, acting as contrast agent for specific pathologies, while at the same time manipulating their interaction with the target in a controlled way. The feasibility of integrating custom MFH systems to existing preclinical MPI scanners has been demonstrated here and previously 30. It can therefore be assumed that integration into clinical systems will be comparatively seamless in the future. The arbitrary confinement of MPI- based MFH to the FFR will enable novel therapeutic options by, e.g., very localized mild and high-temperature hyperthermia to augment radiotherapy and/or chemotherapy.

## Supplementary Material

Supplementary figures.

## Figures and Tables

**Table 1:** Table summarizing the characteristics of the commercial SPION compared in this study with regard to their MFH and MPS performance.

Particle name	Iron oxide core size [nm]	Hydrodynamic diameter [nm]	Coating material	Surface	Product code	Stock concentration [mg(Fe)/mL]
synomag-D-50	20-25	50	dextran	plain	104-00-501	10
synomag-D-70	30-35	70	dextran	plain	104-00-701	10
synomag-S-90	30-35	90	starch	plain	105-00-701	10
synomag-CLD-redF	20-25	50	dextran	plain	125-00-501	3
nanomag-D-spio	5-10	20	dextran	plain	79-00-201	2.4
nanomag-D-spio	5-10	50	dextran	plain	79-00-501	2.4
nanomag-D-spio	10-15	100	dextran	plain	79-00-102	2.4
perimag	5-10	130	dextran	plain	102-00-132	8.5
perimag	5-10	130	dextran	COOH	102-02-132	5
BNF-Dextran	15-20	100	dextran	plain	84-00-102	10

The iron oxide core of the particles is coated with a biocompatible hull (i.e., dextran or starch). The coating of the particles is either plain or conjugated with a carboxyl group (COOH)

## Abbreviations

FFR: field free region
FOT: field of therapy
MFH: magnetic fluid hyperthermia
MPI: magnetic particle imaging
MPS: magnetic particle spectroscopy
SAR: specific absorption rate
SPION: superparamagnetic iron oxide nanoparticles
