# Supplementary material for: In situ theranostic platform combining highly localized magnetic fluid hyperthermia, magnetic particle imaging, and thermometry in 3D
Source: Theranostics. 2024 Jan 1;14(1):324–40. doi: 10.7150/thno.86759 (PMC10750209; doi:10.7150/thno.86759)
Supplement: Supplementary file 1 — Supplementary figures. [file thnov14p0324s1.pdf]

Supplementary Materials for

In situ theranostic platform uniting highly localized magnetic fluid hyperthermia, magnetic particle imaging, and thermometry in 3D

Oliver Buchholz *et al.*

*Corresponding authors Email: oliver.buchholz@uniklinik-freiburg.de
sebastien.baer@uniklinik-freiburg.de

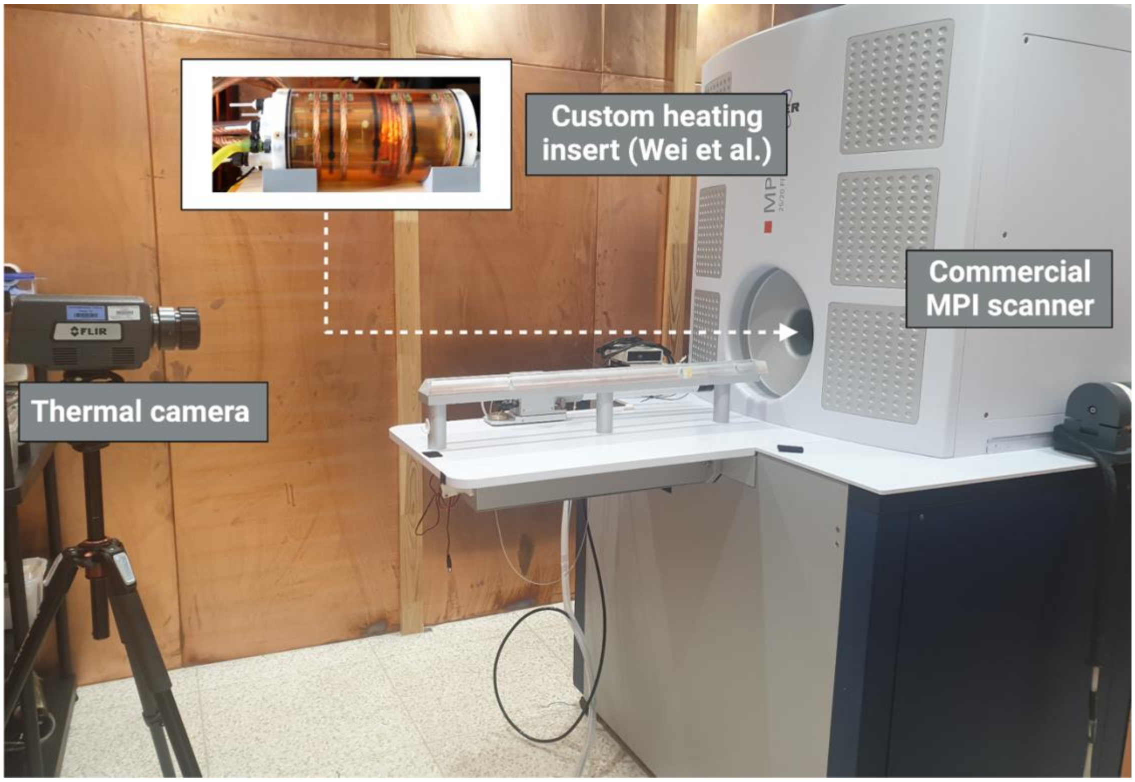

Figure S1: Experimental set-up: A commercial pre-clinical MPI system was equipped with a custom-made heating insert, which was placed in the center of the MPI system's bore. For the experiments described in this study, a thermal camera was positioned approximately 2 meters in front of the MPI system, at the level of the bore.

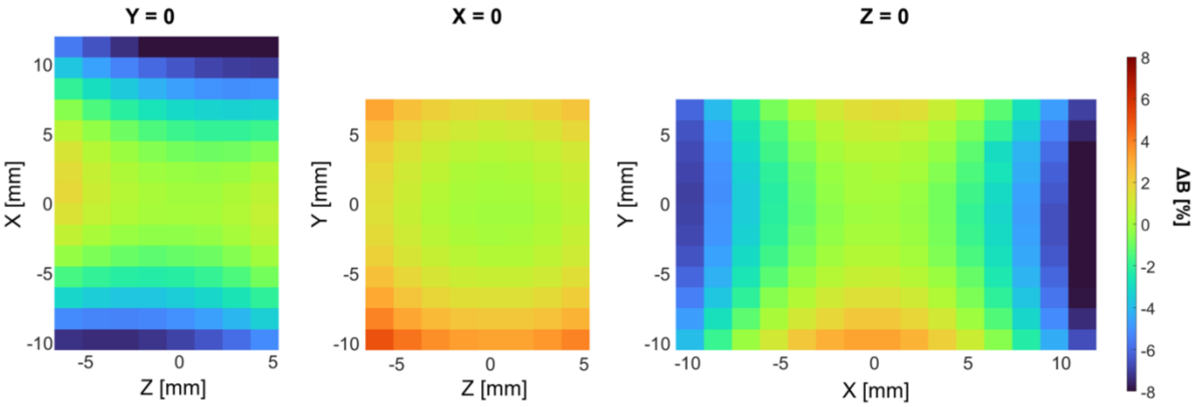

Figure S2: Homogeneity of the custom-made Hyperthermia insert: The plots show the calculated magnetic field strengths of every plane. For the entire field of therapy, a percentage homogeneity of -8.5%, +3.25%, +1.5% (X, Y, Z) were observed.

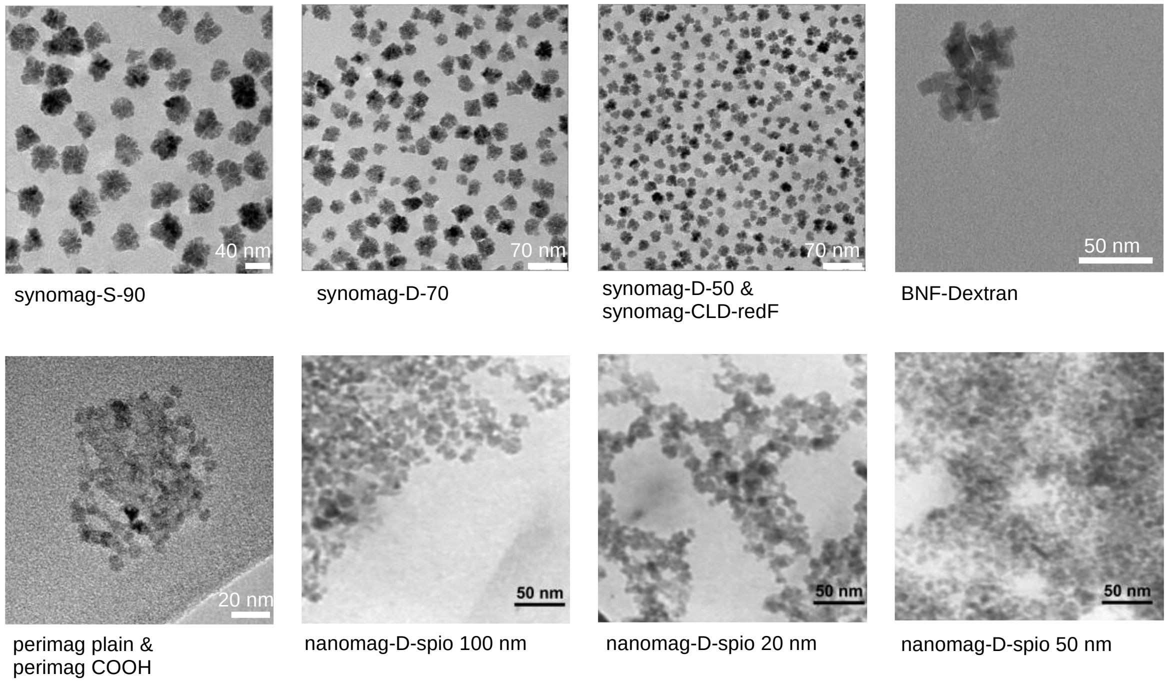

Figure S3: TEM images of the SPION samples used are shown. Some SPION types have the same iron oxide core but differ only in coating, and therefore, they are depicted with a shared image.

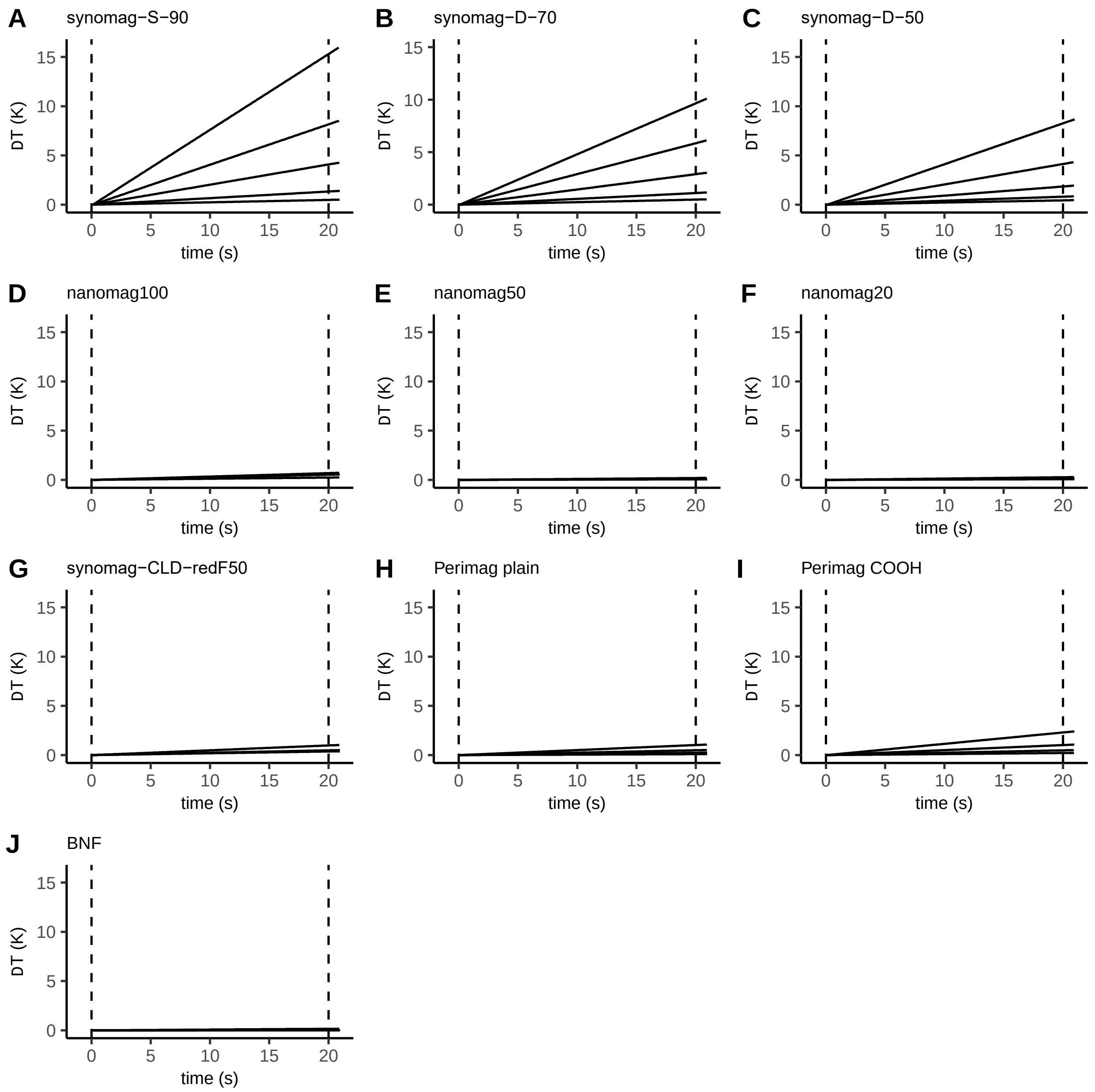

Figure S4: Heating curves of the SPION at different concentrations (0.6, 1.2, 2.4 mg(Fe)/ml for all, 5, and 10 mg(Fe)/ml when applicable) are provided. The individual specific commercial stock concentration served as the upper concentration limit for the respective SPION (see Table 1).

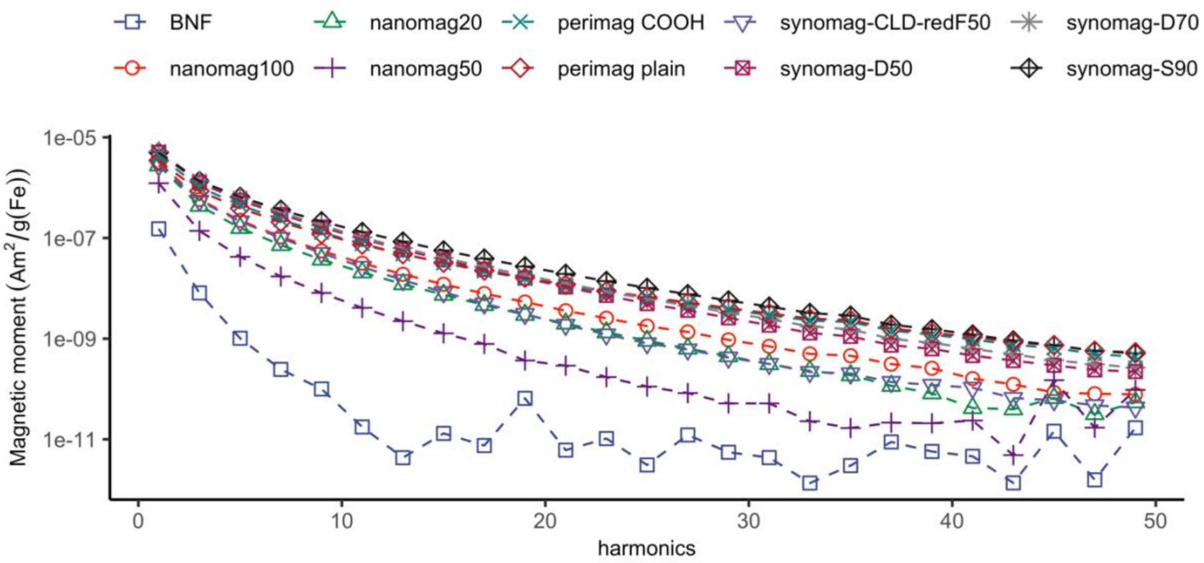

Figure S5: Magnetic particle spectroscopy measurements of all SPION types used in this study (see table 1) were performed and the magnetic moment amplitude for the first 50 harmonics was plotted.

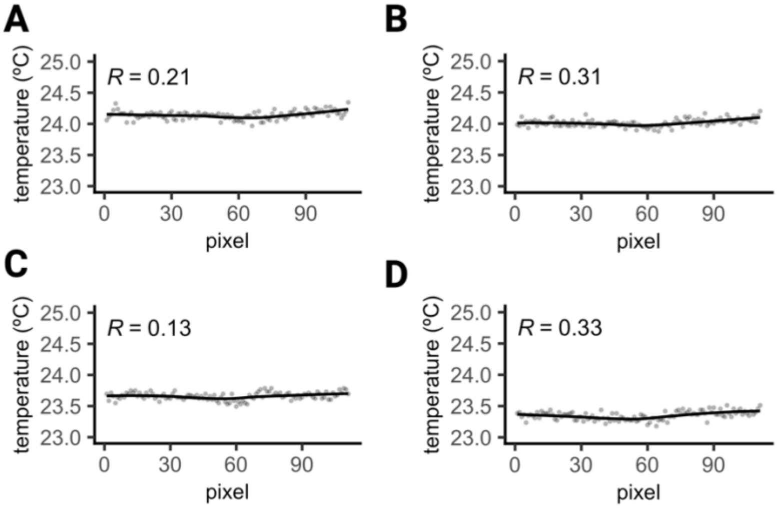

Figure S6: Effect of different flow velocities on heat distribution in a circulation phantom: A tubing system connected to a perfusion pump was utilized to investigate MFH performance under various circulation velocities (see Figure 9 for the experimental setup). The heat distribution of the heated portion of the tube (MFH target) was examined for exemplary velocities. For the shown velocities (0.33 cm/s (**A**), 0.41 cm/s (**B**), 0.83 (**C**) and 6.5 cm/s (**D**)) the temperature is evenly distributed throughout the tube length.
